# Technology agnostic frequency characterization methodology for memristors

**DOI:** 10.1038/s41598-021-00001-6

**Published:** 2021-10-18

**Authors:** Vasileios Manouras, Spyros Stathopoulos, Alex Serb, Themis Prodromakis

**Affiliations:** Centre for Electronics Frontiers, Electronics and Computer Science, University of Southampton, Southampton, SO171BJ UK

**Keywords:** Electronic devices, Characterization and analytical techniques, Electrical and electronic engineering

## Abstract

Over the past decade, memristors have been extensively studied for a number of applications, almost exclusively with DC characterization techniques. Studies of memristors in AC circuits are sparse, with only a few examples found in the literature, and characterization methods with an AC input are also sparingly used. However, publications concerning the usage of memristors in this working regime are currently on the rise. Here we propose a "technology agnostic" methodology for memristor testing in certain frequency bands. A measurement process is initially proposed, with specific instructions on sample preparation, followed by an equipment calibration and measurement protocol. This article is structured in a way which aims to facilitate the usage of any available measurement equipment and it can be applied on any type of memristive technology. The second half of this work is centered around the representation of data received from following this process. Bode plot and Nyquist plot representations are considered and the information received from them is evaluated. Finally, examples of expected behaviors are given, characterizing simulated scenarios which represent different internal device models and different switching behaviors, such as capacitive or inductive switching. This study aims at providing a cohesive way for memristor characterization, to be used as a good starting point for frequency applications, and for understanding physical processes inside the devices, by streamlining the measuring process and providing a frame in which data representation and comparison will be facilitated.

## Introduction

Ever since the introduction of memristors into the electronic device landscape there has been an interest in understanding their internal workings^1^. Over the last decade, multiple characterization schemes have been proposed^2^, all delving into different parameters of memristive devices, from typical benchmarking characteristics, such as, switching dynamics, retention and endurance^3^, to characterizing and exploring the behavior of volatile memristors^4^. Concurrently, many groups are actively trying to make novel devices which will also exhibit variable capacitance and inductance, also known as memcapacitors and meminductors^5,6^, while others use already established technology to create memristive radiofrequency switches^7^. Recently, frequency multiplexing in crossbar arrays has been proposed^8^, were AC signals of different frequencies can be simultaneously supplied to the crossbar and processed semi-independently by frequency band. A cohesive, and generally applicable, methodology is needed in order to encompass all of these types of devices, to quickly pinpoint switching in any of their components and fully characterize their AC behaviour. Furthermore, due to the nature of these devices, electrochemical redox processes are considered to be commonplace^9,10^. Other types of electrochemical processes, such as adsorption–desorption and diffusion, seen more commonly in similar solid state devices, such as, perovskite solar cells^11,12^, lithium-ion batteries^13^, fuel cells^14^, OLEDs^15^, Fe doped SrTiO3 (STO) thin films^16^, may also materialize inside memristors, due to the abundance of oxygen vacancies and other interstitials, thought to be the cause of the resistive switching phenomenon^17^. Many of these processes are better identified in an Alternating Current (AC) environment.

Most characterization methodologies used for memristive systems are carried out with a Direct Current (DC) source, thus making the detection of capacitance and inductance in these devices unlikely, except by indirect inference^18,19^. Furthermore, memristors behave as non-linear electronic devices, with a sensitivity to high voltages, which oftentimes leads to catastrophic failure if not mediated^20^. They also exhibit a non steady behavior with increasing frequency which affects the hysteretic lobe area^21^. Thus simply using alternating polarity between two switching points inserts too much uncertainty to lead to any meaningful result on the actual internal model of the memristor, and any physically important processes taking place inside of it might be overlooked.

The alternative way of testing such a device would be to use a small AC signal superimposed on a DC bias, ensuring that the AC signal stays within a good linear approximation region and does not result in resistive switching. This way capacitive, inductive and diffusive phenomena can be probed, leading to a better understanding of the internal workings of the device.

We propose in this article a "technology agnostic" methodology for testing devices, which, if adhered to, should facilitate the discovery and characterization of the above mentioned phenomena. This will be helpful in gaining a better understanding of memristive devices, while also adding a potent characterization method to the pool of existing methodologies and leading to better comparative studies between proposed technologies.

## Measurement process

### Sample preparation

**Figure 1:**
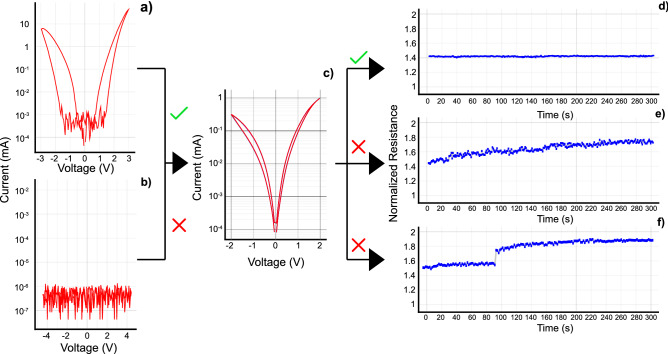
Pristine devices might exhibit a specific signature behavior, declaring them fit for use. Subfigure (**a**) is a representative case of a working pristine device, where a hysteretic loop ,either unilaterally or bilaterally, is an indication of formability. Subfigure (**b**) represents a device which is not available for forming, indicated by the noisy signature. Subfigure (**c**) presents a working device which results from electroforming a device with a typical working pristine signature. Subfigure (**d**) exhibits an acceptable post forming retention test. Devices which exhibit either a volatile behaviour, subfigure (**e**), or sudden instabilities, subfigure (**f**), are discarded as non viable for testing. DC bias for all retention tests was kept stable at 0.5 V.

The protocol for sample preparation (Fig. 1) certifies, through standard DC testing, that any given device exhibits the required stability to be tested under an AC signal. Devices which exhibit faulty or volatile behaviour are discarded in this step, as the inclusion of unstable Devices-Under-Test (DUTs) in a testing process which is expected to create dynamic phenomena will, inevitably, affect in a negative way the results received.

The first step in this selection process is to obtain a pristine IV of the DUT, i.e. a pre-electroforming characteristic, and evaluate it. Currently, one of the categorizations of memristive technologies is in regards of their forming requirements. Most devices need a forming step^22^ but references to "forming free" technologies have also been increasing in recent years^23,24^. For "forming free" devices their pristine IV is tantamount to their memristive IV and thus a normally working "forming free" device can be identified by the typical pinched hysteresis loop. For devices which require a forming step, it is normal for their pristine state to exhibit volatile hysteretic loops either on one or both polarities (Fig. 1a) with increasing voltages, up to reaching a breakdown voltage. These devices need an electroforming step to turn into non-volatile memristors which retain their programmed states. Electroforming can be done either by applying a large bias to the device or by sending a large amount of sequential smaller pulses, with the second method exhibiting a number of advantages^25,26^.

After forming, IV curves (Fig. 1c) of the DUTs must be taken, in order to ensure non-destructive switching in the voltage range that will be used to switch the devices while measuring their frequency response. Most technologies have a measure of behavioural consistency between electroformed devices, and a round of destructive testing is suggested, to ascertain the typical breakdown voltage. This is achieved by submitting some devices to continuously higher switching voltages, until their total breakdown. After receiving this information, a non-destructive switching range can be established. All devices must exhibit the ability to switch in this nondestructive range, before continuing on to the next step. Another key piece of information during this characterization step is to determine a non-invasive testing voltage.

Finally, retention tests are completed to check for volatile characteristics in selected devices (Fig. 1d–f). Volatile behavior can be exacerbated with frequency, if its source is volatile ion movement, and considering its unstable nature, any results received from volatile devices are inherently non-quantifiable, in this framework. A retention test should exhibit stable resistive states sufficiently long to guarantee a maximum desired level of result contamination due to volatility, i.e. on the order of minutes. This is to make sure that the frequency testing takes place inside a theoretically stable state window of the device, in effect enabling the attribution of instabilities to perturbation caused by testing. At the end of the retention test a value of the exhibited resistance should be recorded. Devices that exhibit excessive volatility are to be discarded.

Considering the non-linear nature of memristor I–V curves, as seen in Fig. 1c, it is important to decide on a set DC signal, on top of which the AC signal would be superimposed. This will be the probing voltage. The DC operating point must be the same between equipment used to characterize DC characteristics and AC characteristics, i.e. the DC bias used to characterise nominal resistance readout must be the same as the DC bias on which the AC signal will be superimposed. At the same time, it must be in an area of the IV curve where small changes in bias do not result in big changes in resistance, while also being non-invasive, i.e. not induce switching to the DUT. The AC voltage must be small, to keep the device inside the delimited linear behaviour area. The ultimate amplitude of the superimposed AC voltage is decided by evaluating the results received, very low AC voltage might lead to noisy results.

### Calibration

Calibration is considered of paramount importance to reduce any artifacts or measurement errors coming from the equipment and to receive good quality measurements. Possible sources of erroneous measurements are identified. These are:

- The measuring system itself
- Connections and cables
- Contact with the Devices Under Test (DUTs)
- Problems arising from the device, such as poor adhesion of layers

Before each measurement it is important to follow the same calibration procedure to filter out each of the above sources of error. Equipment for this type of measurements grants the possibility to calibrate both in an open circuit and in a closed circuit, both calibrations are to be made before the next step. Afterwards, a test with a known component should be completed to ensure normal working status of the equipment. If the probing setup requires the usage of probing needles then they should also be filtered out by a second calibration. Through this process, errors arising from the system and the connections are nullified. Contact errors can be corrected by resetting the probes and repeating the measurement a number of times.

Any errors arising by the device itself, in the case where a device made it through the selection process while unfit for testing, can only be attributed to the device when all of the above have been filtered out.

**Figure 2:**
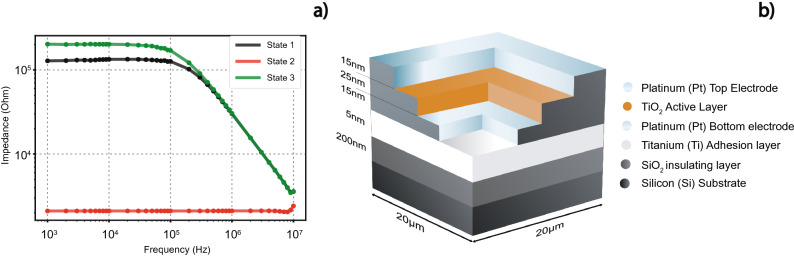
In subfigure (**a**) Typical curves resulting from frequency sweeps are presented. State 1 is the initial measurement, used to locate possible inconsistencies and deviations from expected behaviour. State 2 is the resulting frequency sweep after a switching event, while state 3 is the resulting frequency sweep from a second switching event of the opposite polarity. Subfigure (**b**) depicts the multiple layers which compose the Metal Insulator Metal (MIM) structure of a metal oxide memristor, such as the one used to extract data for Subfigure (**a**).

It is generally advised to develop devices on insulating substrates, thick enough to minimize any stray capacitance which may occur between the electrodes and the substrate. One such solution, if using silicon substrates, might be the deposition of devices on top of a thermally grown film of SiO_2_ as depicted in subfigure (Fig. 2b).

### Measurement protocol

Measurements are mainly done in the form of frequency sweeps. A Keithley 4200-SCS system has been used in this work, but any equipment that can output data as a magnitude and phase angle difference, caused by an AC sinusoidal input, can be used for testing in similar frequencies in the range of 1 kHz–10 MHz. Higher frequency measurements also require the morphology of the device to be tailored in a way that can minimize stray inductance. Thus this part of the protocol will be described with a Keithley equipment, but can be transposed into any other equipment exhibiting a similar range.

In the frequency sweep mode, the output will typically be a bode plot. This is repeated for all the different frequencies inside the sweep. Initially, a frequency sweep of any given device should be done to ascertain the starting state of the electroformed device, before any switching events.

Through repetition this step can become critical in understanding problems with the measurement, such as a faulty contact or a problematic device. Devices of any given technology should exhibit similar behavior.

An example of such a frequency sweep measurement, completed on a metal-oxide memristor, is presented in Fig. 2. The acceptable initial state was characterized by a certain curve as seen in state 1, on Fig. 2. Through repetition of the initial measurement the stability of the device can be confirmed. A switch due to the frequency sweep might mean that the voltage selected is invasive, while a non-repeatable behavior might signify connection problems. The retention step in the sample preparation process ensures that almost all devices have repeatable frequency sweep characteristics.

**Figure 3:**
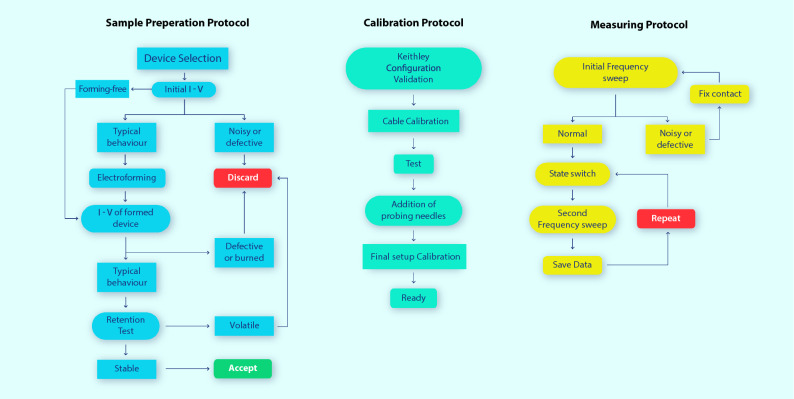
Block diagram representing the decision making process during sample preparation, equipment calibration and measurement protocol. Following the same steps ensures repeatability and limits erroneous measurements.

Following the initial measurement, a voltage sweep can be used to force a switching event inside the device and, subsequently, another frequency sweep to ascertain how this switching affected the device behavior in the tested frequency band, as depicted by states 2 and 3 in Fig. 2. As mentioned at the start of this section, higher frequency measurements necessitate specific device morphology to minimize stray inductance. In state 2, Fig. 2a, a small amount of stray inductance originates from the lines leading from the pads up to the device, producing the apparent small increase at the higher testing frequencies. Switching voltages are selected by consulting the destructive I–V tests, which denote the maximum voltage threshold that a device can accept without leading to catastrophic failure. The final block diagram of the measurement process can be seen in Fig. 3.

## Data representation

### Initial data extraction

Data representation is another important aspect which needs to be streamlined in such a way as to facilitate the comparison of results received from different scientific groups. There have been some examples of possible data representation used in the literature. One such case was the representation in susceptance and conductance^27^ and another is the approach of observing non-zero crossing I–V curves^19^. Both cases suffer from complexity issues and can lead to misrepresentation of raw data by the equipment. In the second case, the information that this method can impart is limited. A commonly accepted characterization process can lead to meaningful comparisons between different technologies, while minimizing risk of data misinterpretation.

We propose, that to characterize a new technology, it is often better to use the raw data extracted by the equipment, as to exclude misrepresentations and internal errors of the measuring equipment. In this case the raw format that the data can be received in is absolute impedance (|Z|) and phase angle (θ). This can then be represented either in two separate bode plots against frequency, or in a Nyquist plot by extracting the real and imaginary part of the total impedance at each specific frequency. Both representation methods have their merits, which will be explored.

### Bode and Nyquist plots

Bode plots can be used to calculate the characteristic cut-off frequency which is derived from the − 3db point of the impedance bode plot. The appearance of more poles or zeros is also evident in this type of plot. Furthermore, these graphs show the actual impedance of any DUT under different frequencies and can be used to understand how such a device would behave inside an actual circuit. In a previous study it was shown, by plotting data received in bode plot format, that some metal oxide memristors behave as discreet RC devices with a programmable resistance, and thus tunable cut-off frequency, while their capacitance is dependent on geometric characteristics of the device^28^.

**Figure 4:**
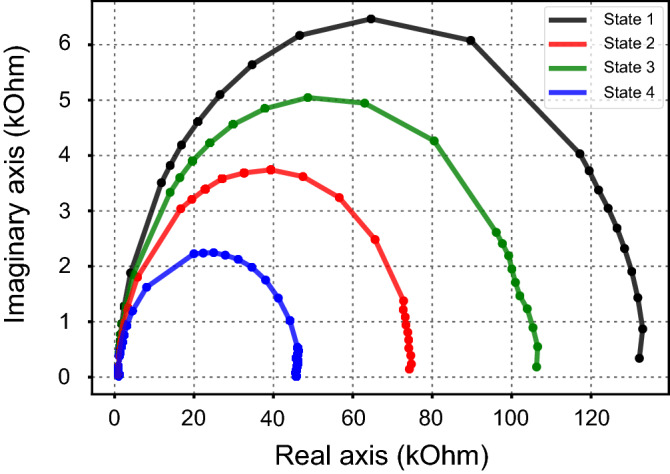
A representation in a Nyquist plot can be useful for identifying the equivalent electrical model corresponding to each technology. It is also useful for calculating capacitance in devices that exhibit RC behaviour. In this type of plot diffusive phenomena are exacerbated and can be easily identified, which is important for devices which have mobile ions in them. Here a device with 4 resistive states is presented, with data experimentally received by following the measurement protocol, applied to a device with layered MIM structure, as shown in Fig. 2b.

A secondary representation of raw data would be in Nyquist plot format as depicted in Fig. 4. This type of data representation is common in fields where devices have mobile ions and processes such as diffusion and adsorption–desorption take place. There is substantial evidence that this might also be the case in many types of memristive systems and thus it constitutes a valid characterization tool to further understand the behavior of these devices, when they are submitted to an AC stimulus.

In Electrochemical Impedance Spectroscopy, devices that exhibit constant phase characteristics are labeled as Constant Phase Elements (CPEs). Other elements were also created for modelling equivalent circuits when liquid or solid electrolytes are present and diffusion phenomena appear. These elements are known as Warburg, finite Warburg, short Warburg and Gerischer elements^29^. In the case of memristors and memristive systems all capacitors can be considered as constant phase elements, CPEs, which in Electrochemical Impedance Spectroscopy behave as part capacitors–part resistors^30^. These CPEs have an impedance calculated by the following equation:

1 $$\frac{1}{Z}=Y={Q}^{o}{(i\omega )}^{n}$$

where ${Q}^{o}$ = $\frac{1}{|Z|}$ while n is a value which defines the characteristics of the CPE.

For n = 1 the above equation becomes the equation of a capacitor and in that case the CPE behaves just like one. For n = 0 the CPE behaves as a resistor. The phase angle of the impedance in this case is independent of frequency, which is where the CPE takes its name from. When a CPE is in parallel with a resistor its Nyquist plot is a semicircle with its center depressed by an angle of (1 − n) × 90° from the X axis of the Nyquist plot.

Most Nyquist plots received from testing these devices are expected to not represent a perfect semicircle, thus making it safe to assume a CPE exists in their actual model. The Nyquist plot is better suited for this type of modelling, since constant phase elements, diffusion dependent elements and ion mobility are more pronounced and the data suggests that electrochemistry plays at least a small role in their switching evolution.

This deviation from an expected capacitive semicircle is commonly attributed to frequency dispersion in electrolyte/electrode systems, which reflects a distribution of time constants in interfacial processes^31^. This behavior has been reported to originate from a number of different parameters, but in this case the most probable one is the roughness of the interface, which has also been proven to be directly correlated to the n value from the CPE equation, where n = 0.5 equals a porous electrode and n = 1 a perfectly flat electrode^32^. Calculation of the n factor can result in valuable data on the state of the interfaces in a memristor and it could indicate possible changes when such a device is switched. This also ties in with prior research carried out on memristive stacks in this laboratory by Michalas et al., where it was discovered that symmetrically constructed devices had asymmetric electrical characteristics^33^. This can be influenced by the circumstances that surround each deposition of materials and lead to different roughness on the interfaces, thus also leading to different “imperfect” capacitors. As interfaces in memristors with a metal–insulator–metal morphology exhibit a roughness which may vary depending on the contact, the extent of the depression can be used to calculate the contact ideality.

Another parameter to consider is the effect of electroforming on the devices. The electroforming process could possibly generate an amount of mobile species inside the material, it can therefore be speculated that a Warburg element, or another adsorption–desorption mechanism, may also be present in the devices. This is reinforced by results received by some device morphologies tested, such as Pt/Al_2_O_3_/TiO_2_/Pt and TiN/TiO_2_/Pt devices, which have at least one non-inert electrode or engineered interface which can function as an ion reservoir. These results will be presented in section III C.

Mobile species, such as, protons, in the form of varying levels of moisture, have been shown to influence memristive performance by altering permittivity values depending on dopant concentration in SiO_2_^34^ or by influencing polarization in memristors based on Polyethylenimine (PEI) film^35^. Thus, any mechanism uncovered by this methodology could be engineered by controlling defects introduced inside the devices.

Modelling of equivalent circuits while using the elements mentioned is not always straightforward and for the adsorption–desorption case, i.e. the low frequency inductive hook appearing in some devices, there does not seem to be any physically valid available model to use. In other fields of study where it is encountered it has still not been fully understood and modelling usually is accomplished by utilizing non physically realized components such as negative capacitors and negative resistors^36^.

Behavior known as low frequency inductive hook has been observed in electrochemical impedance studies in a variety of fields, from fuel cells^37^, to perovskite solar cells^38^, thin films^16^ and studies in corrosion^39^. This behavior is typically associated with intermediate species side reactions^36^, or with oxygen variation in the cathode^40^. The existence of this behaviour in devices tested in this work is another piece of evidence which will be used towards understanding the mechanisms behind the inner workings of memristors. Through increased understanding of these mechanisms the efficiency of fabrication will also be increased.

Lastly, some devices in the literature have a double-layer morphology, but the secondary layer normally is of a few nm thickness. This sometimes raises the question of the actual effect of such a layer on the memristors and in some cases it is speculated that it might not be a cohesive layer, but rather a surface modification. According to the Maxwell–Wagner effect, charge accumulation should be evident at the interface^41^ in these cases. This should lead to a separate time constant for each layer. Absence of this type of behavior may point towards the surface modification layer theory. Electrochemical impedance spectroscopy is a technique which has the ability to point towards specific relaxation times, as they are calculated by the highest point of a semicircle in a Nyquist plot.

## Examples

In the following sections a combination of examples of simulated behaviors or actually measured behaviors with this technique will be presented through the analysis of these proposed cases, the usefulness of the proposed methodology will be established.

### Double layer device morphology

**Figure 5:**
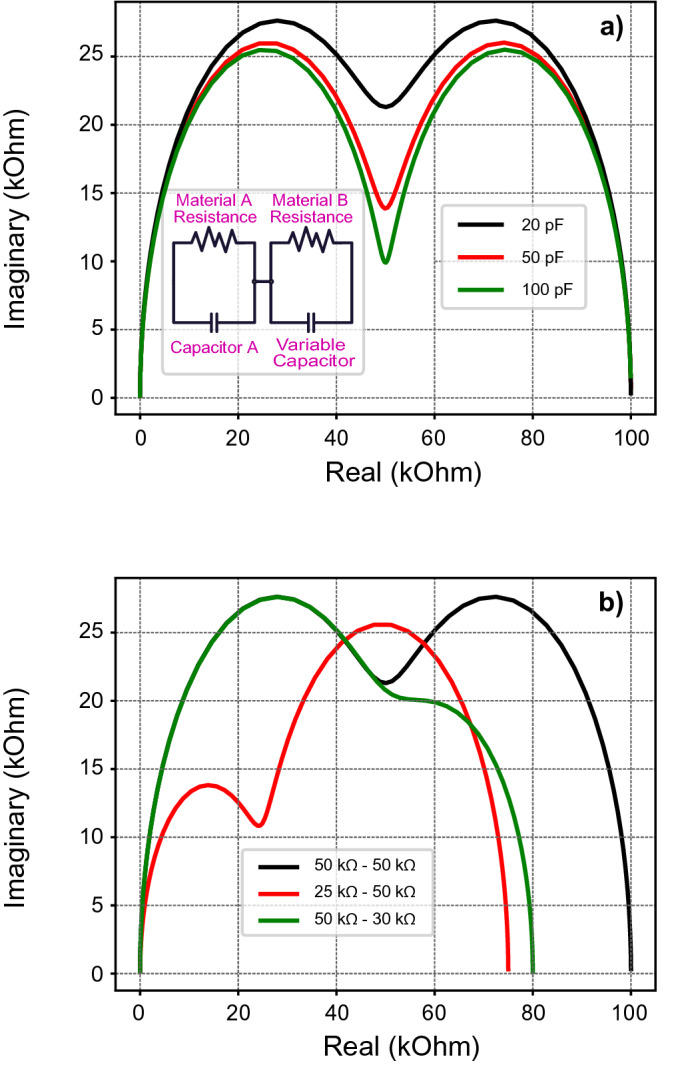
Simulated Nyquist plots of a double layer device, according to the inset equivalent model. In subfigure (**a**), the impact of a changing capacitance in one of the layers is shown. The separation between semicircles is closely related to the ratio of C_1_/C_2_ in this case. Different colour lines correspond to different values in the variable capacitor, shown in the equivalent circuit. In subfigure (**b**), the impact of changing Resistance A or Resistance B is shown. The capacitance values in this case remain stable, with C1 = 1pF and C2 = 20 pF. Here a similar change in resistance does not lead to an equivalent change in both semicircles, as a change in resistance affects the interaction with the capacitor in each RC subcircuit.

A device which consists of two combined active layers could present two semicircles in a Nyquist plot, which makes this type of data representation ideal, as seen in Fig. 5a. In the event where the difference in capacitance of the two layers is high enough, each layer will have a different relaxation time. This will result in charge accumulation due to the Maxwell–Wagner effect and subsequently to the appearance of a secondary semicircle. In Fig. 5a, the simulated characteristic for different capacitive values for one of the layers can be seen. The other layer has a stable capacitance equal to 1 pF in this case. As is apparent, higher ratios of C_1_/C_2_ lead to better defined semicircles.

On the other hand, as seen in Fig. 5b, a change in resistance in any of the two layers leads to a completely different characteristic, while still keeping the double semicircle character intact. With this analysis it is possible to tackle any device of this type and understand where the switching actually takes place. There has been an example of this in the literature^42^. In this case, a device which consisted of two layers is represented as two different semicircles in a Nyquist plot and a switching event changes one of them. This can be considered a strong indication of the switching taking place in only one of the layers in this device.

### Memcapacitor and meminductor

**Figure 6:**
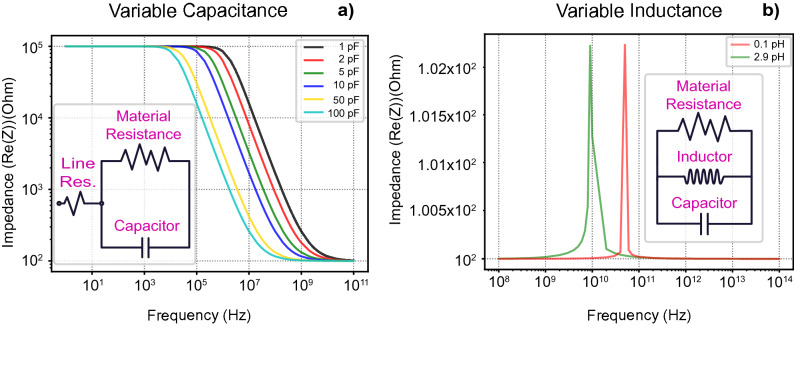
Subfigure (**a**) shows a simulated spectrum of a device with variable capacitance, according to the equivalent model shown in the inset, based with previous results received by our group for metal-oxide memristors. Subfigure (**b**) shows a simulated device with variable inductance, according to the RLC inset, where a variable inductance leads to a shift in the resonant peak.

In our previous work, by following the procedure described here, the internal model of metal oxide memristors has been characterized^28^. This led to an interest in what would be the expected behavior of a metal-oxide memristor which can also change its capacitance. The switch could be due to a variable concentration of mobile ions, such as oxygen vacancies, which might lead to capacitive effects. If the equivalent model remains the same, then a device which presents a variable capacitance would exhibit a behavior close to what is presented in Fig. 6a. In this case a bode plot representation can help identify such a change, where, a device which exhibits capacitive switching, detached from resistive switching, will see its cut-off transported towards a lower frequency, and the slope will be transported parallel to the X axis. A closer approximation of a realizable capacitive device would be one where both capacitance and resistance switch during a switching event. In this case the capacitive change would once again be discernible by quantifying the horizontal transposition of the slope.

On the other hand, for a hypothetical memristive technology which also contains an inductor as part of its internal equivalent model, as seen in the inset of Fig. 6b, a change in inductance would result in a behaviour similar to what is seen in the main part of Fig. 6b. In contrast with the equivalent circuit seen in Fig. 6a, the circuit represented in Fig. 6b has not been confirmed to exist in any actual devices and the final circuit of a device which does behave as a switchable inductor might differ in a number of ways. Changes in inductance in this resonant circuit are expected to transfer the resonance peak towards different frequencies, as seen from the simulated results.

### Ionic motion and diffusion processes inside devices

Lastly, diffusive elements in memristors are a possibility due to their ties to other devices with rich ionic kinetics. Some device morphologies tested during this study, with the aforementioned methodology, have shown a behaviour, known as a low frequency inductive hook, commonly connected with ionic motion^16^ or adsorption–desorption mechanics^13^. This type of behaviour is better identified by using a Nyquist plot, as a departure from an ideal behaviour can quickly be spotted and characterized depending on the observed behaviour. Furthermore, due to extensive research in the field of electrochemistry, an extensive amount of literature exists, to recognize and categorize any emerging diffusive behaviour.

**Figure 7:**
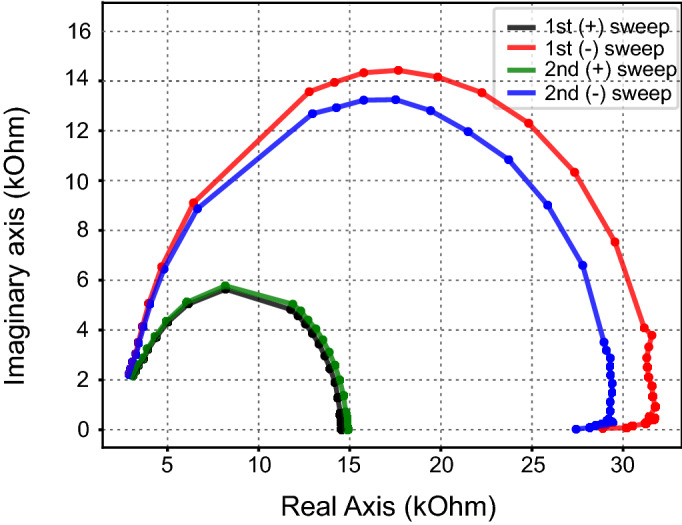
Experimental data measured from a device of TiN/TiO_2_/Pt morphology presents a behavior known as low frequency inductive hook, which appears consistently when a device is switched in higher voltages (− 3 V), only on one polarity, which implies an electrode controlled reaction.

In Fig. 7, data experimentally measured from TiN/TiO_x_/Pt and represented in Nyquist plots show this specific behaviour. This has also been observed in devices with Al_2_O_3_ capping layer. Considering the appearance of this type of behavior only in cases where one electrode is non-inert, or modified with a capping layer, we believe it is directly connected with the adsorption and desorption of vacancies on these layers or the redistribution of vacancies due to the preceding switching event.

Diffusive processes could also be another possible behavior, in lower frequencies. One such possibility would be described by the existence of a Warburg element inside a memristor. Such an element describes a diffusion process in low frequencies which affects the impedance received from the device. The standard equation describing a Warburg element exhibiting a short or finite length diffusion^43^ can be described by the following:

2 $${Z}_{w}\left(\omega \right)=\frac{{W}_{coef}}{{W}_{dif}\sqrt{i\omega }}\mathrm{tanh}\left({W}_{dif}\sqrt{i\omega }\right)$$

where W_coef_ is a coefficient that describes the magnitude of the Warburg behaviour, just as R shows the magnitude of resistive behaviour, which is dependent on material parameters such as electrode area and diffusive species concentration. While W_dif_ is the Nernst diffusion thickness, where W_dif_ = $\frac{d}{\sqrt{D}}$, with d being the diffusion layer thickness and D being the diffusion coefficient of diffusive species.

For this last simulation the EIS Spectrum Analyzer software was used^44^ to simulate the circuit, with an internal model derived from the standard finite Warburg equation described.

**Figure 8:**
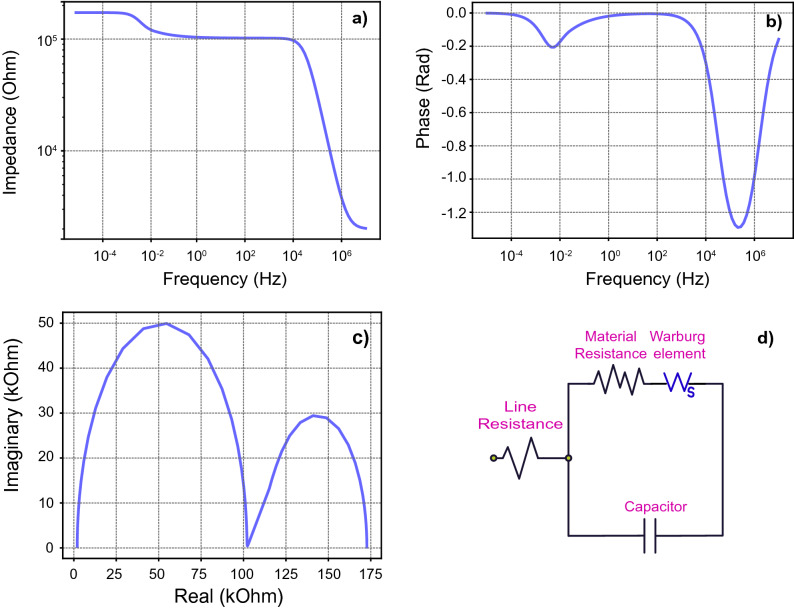
Subfigures (**a**,**b**) represent Bode plots, while, subfigure (**c**) presents the Nyquist plot of a device with an equivalent model which includes a Warburg diffusion element. The equivalent circuit used to simulate the attached plots is presented in subfigure (**d**) and is widely known in electrochemistry as a Randles cell. Data generated with EIS Spectrum Analyzer software^44^.

A memristor with a Randles cell equivalent model, which includes a diffusive Warburg element is presented in Fig. 8. This behaviour while not yet seen in any real devices could evolve into a distinct possibility considering that some active layers could behave as solid state electrolytes, facilitating the diffusion of mobile ions inside the devices.

## Discussion and conclusions

A Warburg element might not yet have been identified inside a working device, but we consider it likely to exist. We believe that the discovery of a diffusion process inside a memristive device could be the missing link to understanding some of the volatile kinetics seen in volatile memristors^4^.

In conclusion, we present a proposed methodology for testing memristive devices. This type of measurement aims at characterizing devices using a small AC signal superimposed on a DC bias. Due to the non-invasive nature of this method it is possible to probe a device without causing switching events. The usage of AC current allows for the discovery of memcapacitive, meminductive and diffusive behaviours in memristors, in a clear and concise way. Furthermore it allows for the creation of an empirical electrical model of a device and can indicate possible ion movement inside it, a detail which could not be emphasized with a typical DC characterization technique. The measurement process was described in detail, from sample preparation to actual measurement protocol, organized in such a way, as to factor out inconsistencies and errors. Preferred methods of data depiction have been covered, briefly illuminating the usefulness of each type of representation. Lastly, examples of actual data received through this method and simulated data were shown. In double layer devices this method elucidates where the switching takes place, thus clarifying the importance of each layer in multilayered devices. Simulated data on memcapacitors and meminductors show a glimpse on how these devices would behave when characterized using this technique. Finally, both experimental data from ion mobility inside devices and simulated data from diffusive processes have been presented, which can be helpful in further understanding the internal structure of these devices. We believe that this work will add a valuable tool in existing memristive characterization routines, leading to a better understanding of existing memristors and to a more organic approach in the design of future devices.

## Data Availability

All data used in plotting the figures are available at 10.5258/SOTON/D1988
